# Life-course socio-economic status and adult BMI in Ghana; analysis of the WHO study on global ageing and adult health (SAGE)

**DOI:** 10.1186/s12939-016-0474-x

**Published:** 2016-11-15

**Authors:** Tomi F. Akinyemiju, Xueyan Zhao, Swati Sakhuja, Pauline Jolly

**Affiliations:** 1 Department of Epidemiology, University of Alabama at Birmingham, Birmingham, AL 35294-0022 USA; 2 Comprehensive Cancer Center, University of Alabama at Birmingham, Birmingham, AL USA

**Keywords:** BMI, Ghana, Life-course, Obesity, Socioeconomic status

## Abstract

**Background:**

Obesity rates have continued to increase over time globally, resulting in an increase in the burden of obesity-associated chronic diseases. There is a paucity of research on the association between obesity and generational changes in socio-economic status (SES) in developing countries like Ghana, and therefore a critical need to better understand within-country differences in obesity and its association with SES over the life-course.

**Methods:**

Data from a nationally representative sample of adult women in Ghana was used to examine the association between life-course SES and adult body mass index (BMI). Life-course SES was defined based on changes in the employment and education status of both parents and the study participant. Survey weighted multivariable linear regression models were used to examine the association between individual and life-course SES in relation to BMI.

**Results:**

Participants with higher SES over their life course, that is, both the participant and her father had at least a primary education (both > = primary vs. both < primary: BMI 27.2 vs. 24.1), and both were employed (both employed vs. both unemployed: BMI 26.5 vs. 24.4) had higher BMI compared with participants with lower SES over their life course.

**Conclusion:**

Higher individual and life-course SES is associated with higher BMI among women in Ghana, although maternal employment was associated with lower BMI.

## Key messages

- By 2030, up to 70% of all non-communicable disease cases will occur in developing countries, however this figure is likely to be higher given the accelerated increase in obesity rates.
- Lower SES individuals in developed countries experience higher obesity rates due to lower access to affordable, healthy food compared instead of highly processed and cheaper fast food options.
- Higher SES individuals in developing experience higher obesity rates likely due to the recent influx of ‘western style’ fast food high in fat and refined carbohydrates, and a decline in traditional diets rich in fruits, leafy vegetables, and unrefined carbohydrates.
- In Ghana, there is a strong influence of SES over the life-course on adult BMI among women

## Background

Renewed attention has been focused on the negative health impacts of obesity, with multiple studies providing evidence that the risk of developing and dying from non-communicable diseases is significantly higher among obese compared with non-obese individuals [1–4]. The relative risk of developing diabetes, coronary heart disease, stroke, hypertension, and multiple cancers is much higher for overweight/obese individuals (Body-mass index or BMI >25 kg/m^2^) compared with normal weight individuals [4]. Although the global prevalence of obesity has risen rapidly in the past 3 decades - increasing from 29 to 38% in women - the rate of increase in developed countries has slowed, while obesity rates continue to accelerate in developing countries [5]. For instance, recent studies report overweight/obesity prevalence of almost 30% among young adults in developing countries [6], and prevalence of overweight/obesity exceeded 20% in 92% of developing countries worldwide [7]. The World Health Organization predicts that by 2030, up to 70% of all new non-communicable disease cases will be observed in developing countries due to population size and ageing [8]. If present trends in obesity rates continue, the actual burden of cases will be significantly higher, as most developing countries remain ill-prepared to handle the unprecedented growth in the burden of non-communicable diseases [9].

Changes in obesity rates over centuries have been attributed to advances in modern agriculture and economic development [10, 11]. The ‘nutritional transition’ describes changes in food consumption patterns that occur as population groups move from hunter-gatherers (characterized by a diet of leafy green vegetables, fiber and unrefined carbohydrates, and high physical activity) to modern agriculture and receding famine (associated with a diet high in total fat, cholesterol, and refined carbohydrates and a more sedentary lifestyle), and the eventual behavioral change back to a diet of high fruit, vegetable and complex carbohydrates consumption and increased physical activity [12]. These transitional stages have been well documented in developed and developing countries; developed countries have transitioned through these stages over centuries and are now mostly at the behavioral change phase [13]. In contrast, economic development, modernization of farming and globalization in developing countries have recently begun to spur a nutritional transition from traditional diets to diets high in total fat, cholesterol and refined carbohydrates [12]. The impact of these changes on rising obesity rates in developing countries is becoming more apparent.

Although the prevalence of obesity varies significantly between developed and developing countries based on stage of transition, significant differences also exist within countries. There is a well established inverse relationship between socio-economic status (SES) and obesity in developed countries such as the United States, where low SES individuals are less likely to have access to, or afford, healthy diets including fresh fruits and vegetables, compared to highly processed fast food high in fat and cholesterol [12], leading to higher prevalence of obesity. The association between SES and obesity in developing countries appears to be the reverse; with studies reporting that more affluent or those with higher education were more likely to be obese [14, 15]. Additionally, studies from developed countries suggest that the influence of SES on obesity begins from childhood [16–19]. However, very few studies have focused on examining socio-economic differences across the life-course in obesity in developing countries, and to our knowledge only one study has been conducted in sub-Saharan Africa to examine childhood and adult SES. Addo et al. failed to observe an association between pre-adult wealth (using durable household assets as an indication of wealth) and adult BMI among civil servants, but did observe that older women with more current wealth were more likely to be obese, whereas younger women with more current wealth were less likely to be obese compared to their less wealthy counterparts [18]. This suggests that there may be a generational change in the influence of SES on obesity, consistent with different stages of the nutritional transition among population groups within the same country. Studies examining both childhood and adult SES in relation to obesity may help to better characterize this phenomenon.

By understanding within-country differences in obesity and association with SES over the life-course, obesity prevention efforts can be focused on the population subgroups at highest risk. In this study, we examine the association between life-course SES on adult BMI using employment and education status of parent and adult child as concrete measures of SES among a nationally representative sample of women in Ghana.

## Methods

Data for these analyses were obtained from Wave 1 of the Ghana Study of Global Ageing and Health (SAGE) conducted in 2007. The SAGE study was organized by the World Health Organization, and designed as a longitudinal study focused on adults ages 18 and older from nationally representative samples in China, Ghana, India, Mexico, Russian Federation and South Africa. The survey was designed to assess the health status and well being of adult populations by focusing on individual and household level variables. Further information on the SAGE study including details about the sampling methodology can be obtained through the SAGE website: (http://www.who.int/healthinfo/sage/cohorts/en/).

Our sample consisted of a total of 2341 women ages 18 years and older surveyed as part of the Ghana SAGE study. Participant demographic characteristics were examined and paternal and maternal education variables were each categorized into no formal education, primary school only, high school graduate, and college or higher degree. Participant, paternal and maternal employment variables were each categorized into unemployed, public sector employment, private sector employment, self/informal employment. In addition, we determined life-course SES based on changes between paternal, maternal and participant education and employment. Life-course SES based on parental and participant education was categorized into: < primary and < primary; > = primary and < primary; < primary and > = primary; > = primary and > = primary, and life-course SES based on parental and participant employment was categorized into: unemployed and unemployed; unemployed and employed; employed and unemployed; and employed and employed. Body mass index was calculated based on height and weight measurements taken by the interviewer during the questionnaire administration process.

### Statistical analysis

We conducted simple descriptive analysis with means and standard errors to determine the distribution of participants’ BMI by participant and parental SES, as well as by life-course SES measures. Multivariable linear regression models were created to study the relationship between BMI and SES as well as life-course SES. Furthermore, regression models were used to analyze the associations adjusted for age, smoking, alcohol, health status, residence, and marital status. Adjusted means for BMIs were calculated using least-squares options of the survey linear regression procedure in SAS using survey weight, strata and cluster variables provided with the SAGE dataset to account for study sample design and enabling study results to be generalizable to the entire country. For all analyses, *p* values ≤ 0.05 were considered statistically significant. All statistical analyses were performed with SAS 9.4 (SAS institute Inc. NC, USA).

## Results

In unadjusted analysis (Table 1), participants own education and employment status was significantly associated with BMI (*p*-value: 0.0182 and 0.0253, respectively) with the highest BMI for those who were self-employed compared to those who were employed in the private sector (27.8 vs. 24.1). Father’s education and both parents’ employment status was significantly associated with BMI (*p*-values < 0.05). Women whose fathers had at least a college degree had the highest BMI compared with women whose fathers had no formal education on average (BMI: 28.1 vs. 24.7). There was no significant difference in BMI by participant’s mother education. Similar trends were observed for employment status; the highest BMI was observed among women whose fathers were employed in the public sector compared with those whose fathers were unemployed (father: 28.7 vs. 22.6).

**Table 1:** Unadjusted mean BMI and socio-economic status, 2008 WHO SAGE

	N (%)	BMI Mean (SE)	*P*-value
Overall	2341	25.27 (0.3)	
Age (in years)			**<0.0001**
< 21	18 (2.7)	25.12 (2.4)	
21-39	180 (39.6)	25.32 (0.5)	
40-64	1167 (47.3)	25.73 (0.5)	
> = 65	976 (10.5)	22.97 (0.3)	
Marital Status			0.5691
Never Married	65 (8.9)	24.40 (0.8)	
Married/cohabiting	779 (58.8)	25.33 (0.4)	
Sep/Div/Wid	1497 (32.3)	25.38 (0.7)	
Smoking			0.4410
No	2166 (94.5)	25.32 (0.3)	
Yes	175 (5.5)	24.26 (1.3)	
Alcohol use			0.9796
No	2098 (88.8)	25.26 (0.3)	
Yes	243 (11.2)	25.30 (1.8)	
Health Status			0.4865
Bad	543 (25.6)	24.73 (0.5)	
Moderate	1001 (30.4)	25.79 (0.7)	
Good	797 (44.0)	25.22 (0.5)	
Education			
Own			
No formal education	1675 (53.5)	24.32 (0.5)	**0.0182**
Primary school	248 (16.4)	26.28 (1.1)	
High school	357 (27.0)	26.42 (0.5)	
College or more	51 (3.4)	26.15 (1.0)	
Mother’s			
No formal education	2219 (89.2)	25.11 (0.4)	0.3062
Primary school	38 (4.3)	27.98 (1.9)	
High school	49 (5.7)	25.54 (1.0)	
College or more	8 (0.8)	27.56 (2.1)	
Father’s			
No formal education	1912 (71.7)	24.69 (0.5)	**0.0030**
Primary school	89 (6.2)	25.69 (1.1)	
High school	210 (16.5)	26.99 (0.7)	
College or more	44 (5.5)	28.12 (1.0)	
Employment			
Own			
Unemployed	716 (17.5)	24.09 (0.6)	**0.0253**
Self/Informal employment	1526 (76.5)	27.79 (1.7)	
Public sector	64 (3.7)	25.39 (0.4)	
Private sector	35 (2.2)	26.67 (0.7)	
Mother’s			
Unemployed	93 (4.5)	26.01 (1.4)	**0.0121**
Self/Informal employment	2211 (92.1)	25.17(0.4)	
Public sector	26 (2.8)	27.90 (1.7)	
Private sector	11 (0.6)	22.08 (1.1)	
Father’s			
Unemployed	53 (2.6)	22.57 (1.1)	**0.0011**
Self/Informal employment	2019 (76.3)	24.54(0.3)	
Public sector	224 (17.5)	28.72(1.2)	
Private sector	45 (3.6)	25.83(1.0)	

Bold indicates significance *p* value ≤ 0.05

In addition, life-course SES based on participant and father’s education and employment, but not mother’s education and employment, was significantly associated with BMI (*p*-value: 0.015 and <0.0001, respectively, Table 2). Participants with higher SES over their life course, that is, both the participant and her father had at least a primary education (both > = primary vs. both < primary: BMI 27.2 vs. 24.1), and both were employed (both employed vs. both unemployed: BMI 26.5 vs. 24.4) had higher BMI compared with participants with lower SES over their life course.

**Table 2:** Unadjusted mean BMI and life-course socio-economic status, 2008 WHO SAGE

Life-course SES		N	BMI (SE)	P-value
Education				
Mother’s	*Own*			0.12
< Primary	< Primary	1664	26.75 (1.1)	
> = Primary	< Primary	21	26.26 (0.6)	
< Primary	> = Primary	582	26.30 (1.5)	
> = Primary	> = Primary	74	24.26 (0.5)	
Father’s	*Own*			**0.015**
< Primary	< Primary	1584	24.11 (0.5)	
> = Primary	< Primary	101	25.99 (0.9)	
< Primary	> = Primary	414	25.67 (0.7)	
> = Primary	> = Primary	242	27.21 (0.6)	
Employment				
Mother’s	*Own*			0.07
Unemployed	Unemployed	2216	25.11 (0.4)	
Employed	Unemployed	26	27.10 (2.0)	
Unemployed	Employed	88	27.27 (0.7)	
Employed	Employed	11	26.55 (2.3)	
Father’s	*Own*			**<0.0001**
Unemployed	Unemployed	2006	24.36 (0.3)	
Employed	Unemployed	236	28.56 (1.2)	
Unemployed	Employed	66	27.88 (0.9)	
Employed	Employed	33	26.51 (1.1)	

Bold indicates significance *p* value ≤ 0.05

After adjusting for age, smoking, alcohol, health status, residence, and marital status (Table 3), employment status of both parents (*p*-value: 0.026 and 0.0003 for mother and father, respectively), and education status of both the participant and her father (*p*-value: 0.024 and 0.014, respectively) were each significantly associated with BMI. Participants whose mothers were employed in the public sector had significantly higher BMI compared with those who were unemployed (24.7 vs. 22.4); and participants whose fathers were employed in the public sector had higher BMI compared with those whose fathers were unemployed (25.3 vs. 19.00). In the fully adjusted model including other SES variables, only mothers’ and fathers’ employment status remained significant. Participants with mothers who were employed in the public sector had lower BMI compared with those whose mothers were unemployed (23.5 vs. 25.31). Conversely, those whose fathers were employed in the public sector had higher BMI compared with those whose fathers were unemployed (26.51 vs. 19.53).

**Table 3:** Multiple regression models of adjusted mean BMI and socio-economic status

Socio-economic status	Adjusted BMI^a^	*P*-value	Fully adjusted BMI^b^	*p*-value
Education				
Own		**0.0242**		0.4305
No formal education	21.49	0.03	22.04	0.46
Primary school	23.24	0.79	23.98	0.58
High school	23.39	0.85	22.82	0.87
College	23.58	-	23.05	-
Mother’s		0.3772		0.6727
No formal education	21.75	0.20	22.28	0.50
Primary school	24.13	0.91	23.43	0.73
High School	22.32	0.35	21.35	0.36
College	24.43	-	24.84	-
Father’s		**0.0138**		0.8431
No formal education	21.70	0.0051	23.48	0.83
Primary school	22.43	0.11	22.23	0.75
High School	23.74	0.36	23.28	0.83
College	24.93	-	22.91	-
Employment				
Own		0.0903		0.1212
Unemployment	21.09	0.037	21.86	0.03
Self/informal	21.92	0.09	23.03	0.14
Public	23.13	0.40	21.68	0.06
Private	24.58	-	25.32	-
Mother’s		**0.0261**		**0.0033**
Unemployment	22.41	0.046	25.31	0.0006
Self/Informal	21.59	0.040	23.08	0.0051
Public	24.70	0.0035	23.50	0.09
Private	18.34	-	20.00	-
Father’s		**0.0003**		**0.0189**
Unemployment	19.00	0.012	19.53	0.04
Self/Informal	21.27	0.14	22.61	0.68
Public	25.26	0.16	26.51	0.18
Private	22.93	-	23.24	-

^a^Adjusted for age, smoking, alcohol, health status, residence, and marital status

^b^Adjusted for age, smoking, alcohol, health status, residence, marital status, own education, mother’s education, father’s education, own employment, mother’s employment and father’s employment

Bold indicates significance *p* value ≤ 0.05

**Fig. 1:**
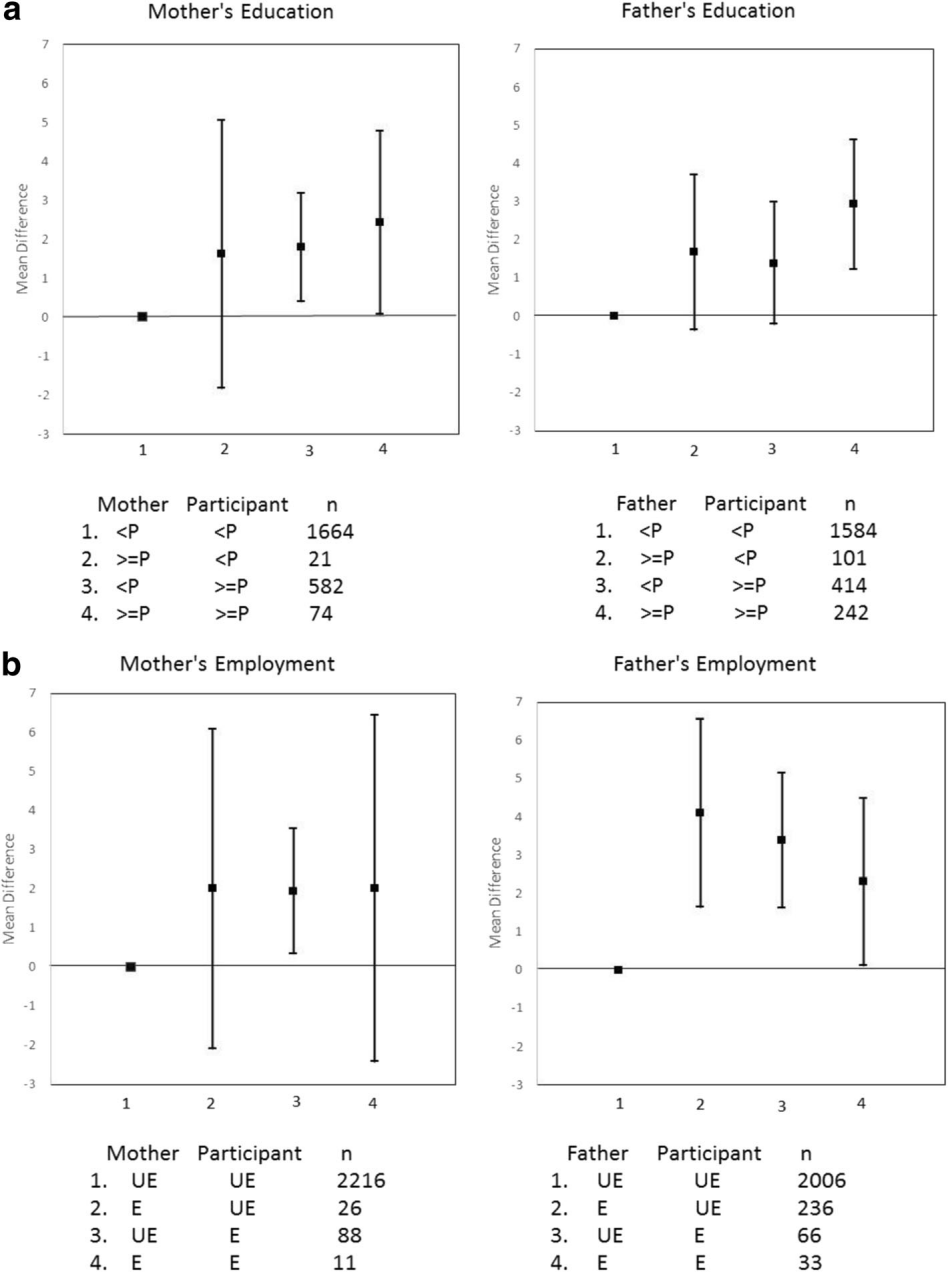
Adjusted mean difference in BMI (95 % confident interval) for life-course Socio-economic status trajectory comparing mother’s and father’s education (<*p*: less than primary; >=*p*: at least primary school education) with participant’s education status **a**, and mother’s and father’s employment (E: employed, UE: unemployed) with participant’s employment status **b**. Models were adjusted for age, smoking, alcohol, health status, residence, and marital status

In the adjusted model (Table 4), life-course SES based on both maternal and paternal education and paternal employment were each associated with BMI (*p* < 0.05). Compared with participants with low education and parents with low education, participants with high life-course SES based on education had higher BMI. For instance, participants with low life-course SES based on maternal education had on average 2.4 points lower BMI compared with high life-course SES based on maternal education (21.5 vs. 23.9). There was no significant association with life-course SES based on mother’s employment, although participants who had high life-course SES based on paternal employment had significantly higher BMI compared with those who had low life-course SES based on paternal employment (BMI: 23.5 vs. 21.2). The largest BMI difference was observed between those who had low life-course SES based on paternal employment, (that is, they were unemployed and had fathers who were unemployed, and those who experienced downward mobility based on employment (that is, their father was employed but they were unemployed) (BMI: 21.2 vs. 25.3). Figure 1 illustrates the adjusted results showing trends in BMI by life-course SES. There was a positive trend of higher BMI with increasing life-course SES based on maternal and paternal education, but an inverse trend of decreasing BMI with increasing life-course SES based on paternal employment.

**Table 4:** Multiple regression models of adjusted mean BMI and life-course socio-economic status

Socio-economic status		Adjusted BMI^a^	*p*-value
Education			
Mother’s	*Own*		**0.0339**
< Primary	< Primary	21.48	-
> = Primary	< Primary	23.10	0.36
< Primary	> = Primary	23.27	0.01
> = Primary	> = Primary	23.90	0.04
Father’s	*Own*		**0.0062**
< Primary	< Primary	21.45	-
> = Primary	< Primary	23.13	0.11
< Primary	> = Primary	22.84	0.09
> = Primary	> = Primary	24.39	0.0007
Employment			
Mother’s	*Own*		0.0852
Unemployed	Unemployed	21.63	-
Employed	Unemployed	23.64	0.33
Unemployed	Employed	23.58	0.02
Employed	Employed	23.65	0.37
Father’s	*Own*		**< .0001**
Unemployed	Unemployed	21.17	-
Employed	Unemployed	25.28	0.001
Unemployed	Employed	24.57	0.0002
Employed	Employed	23.48	0.039

^a^Adjusted for age, smoking, alcohol, health status, residence, and marital status

Bold indicates significance *p* value ≤ 0.05

## Discussion

In this study, we evaluated the association between life course SES and BMI among adult Ghanaian women using parental education and employment to assess childhood SES, and participant education and employment to assess SES in adulthood. Mother’s employment was the only variable independently associated with decreased BMI among participants. Although BMI was lowest among participants who were unemployed and whose fathers were unemployed, BMI was also lower when both were employed compared with when only one person was employed. Increasing levels of all other SES variables, both parental and individual, were associated with increasing BMI among participants. These findings are consistent with other studies showing that at the initial stages of economic development, higher SES is associated with higher BMI [13, 20, 21]. However in later stages, the association became reversed, as higher SES individuals gain increased awareness of the negative health consequences of obesity. This likely explains our findings of a normal distribution for mean BMI: lower when both father and daughter are unemployed; higher when either one is unemployed; and lower again when both father and daughter are employed.

Our finding that fathers’ employment and education were a strong and consistent predictor of participant BMI may be explained by the fact that in many developing countries such as Ghana, men are still the primary source of income for the household. Therefore, the SES of the entire household is directly linked to the income and education status of the father. However, we also observed that participants whose mothers were employed in either the private or public sector had lower BMI compared with those whose mothers were unemployed, regardless of fathers’ employment and education. This strongly suggests that mothers’ employment status also plays a critical role in shaping women’s early life exposure to diet and nutrition, and possibly shapes perceptions about body size and health as well. There are several possible explanations for this: 1) mothers’ employment may provide extra household income for healthier food options; 2) employment outside the home may increase exposure to health information regarding healthy body weight, especially if the employment includes health insurance and hence access to medical personnel; 3) employment outside of the home may also lead to greater physical activity for mothers, especially if the work involves physical labor, thereby influencing daughters’ perceptions about ideal body size. These factors have been shown to be important in determining childhood obesity rates [22–24], and although employment outside of the home may potentially negatively influence dietary habits in children, our observation of an inverse association between maternal employment and offspring BMI does not support this hypothesis.

Cultures and traditions are likely to change significantly as globalization reduces socio-cultural boundaries between countries. These changes are associated with widespread availability of ‘western-style’ fast food options in many urban cities in developing countries, direct exposure to mass media advertisements, and shifts in occupational patterns from labor intensive farming and agriculture to highly sedentary service and professional sectors [10]. The past couple of decades have also been accompanied by unprecedented economic development in developing countries, increasing the purchasing power of individuals who could previously not afford Western food items. Unless targeted obesity prevention strategies are employed, a lifestyle of traditional diets with high physical activity may continue to erode, leading to further increases in BMI. Our results suggest that higher SES individuals in Ghana may have been first to adopt these lifestyle changes, as higher SES individuals have higher BMI compared with other groups. However, they may also be more likely to quickly obtain access to health information regarding the health implications of obesity and transition into a healthier lifestyle, while lower SES individuals may experience increasing BMI as the previously ‘foreign’, ‘exotic’ and expensive food items become cheaper [25]. There is a need for public health strategies designed to reverse this trend as the under-developed and under-resourced healthcare and public health infrastructure in many developing countries like Ghana may be unable to handle the influx of obesity-associated chronic diseases.

There are several strengths of this analysis. First, we were able to use recent data from the World Health Organization, collected in a standardized format and representative of the entire country of Ghana. In addition, BMI was measured by trained study interviewers and not self-reported, improving the reliability of the measurement. Furthermore, we used employment and education status variables to assess SES for parents and participants as these variables are not likely to be influenced by serious recall bias. Furthermore, since the survey was designed to assess a wide-range of health issues, we do not anticipate differential recall bias by BMI status. There are also a few limitations. First, we were unable to assess household income directly in the dataset, although we expect that education and employment status will provide a more reliable measure of SES, especially in situations where compensation may be ‘in-kind’ as opposed to cash. Second, the survey was conducted 7 years ago and estimates observed here may have changed. However, we anticipate that given the positive trends in World Bank economic projections for Ghana, suggesting that the economy has improved significantly, our results are likely to be under-estimates rather than over-estimates of current BMI.

## Conclusion

In conclusion, higher individual and life-course SES is associated with higher BMI among women in Ghana, although women whose mothers were employed had lower BMI. Public health efforts may focus on providing information on obesity prevention strategies to high SES individuals and their children, and national strategies focused on encouraging consumption of healthier, traditional dietary patterns rich in unrefined grains and leafy vegetables may go a long way in reducing the burden of obesity and obesity-associated chronic diseases in the coming decades.

## Abbreviations

BMI: Body-mass index
SAGE: Study of Global Ageing and Health
SES: Socio-economic Status
WHO: World Health Organization
